# Supplementary material for: Understanding thromboembolus transport patterns in the brain for stroke in the presence of carotid artery stenosis
Source: PLoS Comput Biol. 2025 Sep 8;21(9):e1013269. doi: 10.1371/journal.pcbi.1013269 (PMC12533973; doi:10.1371/journal.pcbi.1013269)
Supplement: S1 Text — (PDF) [file pcbi.1013269.s001.pdf]

Understanding Thromboembolus Transport Patterns In The Brain For Stroke In The Presence Of Carotid Artery Stenosis

Ricardo Roopnarinesingh, Neel D. Jani, Michelle Leppert, Debanjan Mukherjee

Supplementary Material Information

S1 Supporting Information Legend

S1_Text.pdf: *Supplementary information pdf file:* compilation of additional modeling information, data, and detailed description of videos/animations provided with the manuscript.

S1_Movie.mp4: *Animation/Movie:* showing the source to destination embolus transport for emboli originating from left/right carotid stenosis and cardiogenic sources, for one of the bilateral stenosis models.

S2_Movie.mp4 *Animation/Movie:* showing contralateral embolus movement across the arterial network of the Circle of Willis for a 40% right carotid stenosis and 85% left carotid stenosis combination.

S3_Movie.mp4 *Animation/Movie:* showing contralateral embolus movement across the arterial network of the Circle of Willis, varying across different combinations of stenosis severity as indicated in the video header.

S4_Movie.mp4 *Animation/Movie:* showing the transport mechanics of emboli from cardiogenic sources compared against emboli from carotid stenosis sites, for CCO cases.

S5_Movie.mp4 *Animation/Movie:* showing local hemodynamic patterns including jet flow across the stenosis throat for varying stenosis severities.

S6_Movie.mp4 *Animation/Movie:* showing proximal collateral circulation variations with varying stenosis in the brain.

S2 Animation of stenosis flow and embolus dynamics

Here, we describe the simulation animations that have been provided as additional illustration of the simulation data to support discussions presented in the main manuscript.

Animation titled: ***S1_Movie.mp4*** shows how all three embolus releases travel into the cerebral arteries from respective surface, presented for the non-CCO 40% stenosed right carotid with 85% stenosed left carotid case. Dynamic source-to-destination transport patterns, trends in embolus recruitment in the vertebrobasilar pathway, and contralateral movement into the anterior CoW vessels can bee seen in the video.

Animation titled: ***S2_Movie.mp4*** shows one instance of how contralateral trans-hemispheric embolus movement can be enabled through the AcoA, with emboli moving from the right carotid release to the left hemisphere. This for the case of 10% right carotid stenosis paired with 85% left carotid stenosis.

Animation titled: ***S3_Movie.mp4*** shows thromboemboli released from two sources, both from the right carotid but of differing stenosis severity. This animation illustrates how the likelihood of contralateral movement is higher in cases with higher severity pairing where the amount of particles traveling into the left hemipshere is visibly higher in the model with a 40% right severity and a 85% left severity.

Animation titled: ***S4_Movie.mp4*** compared two differing cases of CCO with one having a 10% severity and the other 85%. Both the cardiogenic and carotid releases are shown where we can note two distinct mechanics: (1) the recruitment of cardiogenic embolus through the vertebral arteries that supplements the occluded and highly stenosed carotid arteries, (2) the distinctly different hemodynamics within the 85% stenosed carotid where the embolus movement is much more erratic and less intuitive due to lack of flow and the jet impingement.

Animation titled: ***S5_Movie.mp4*** shows the flow disturbance caused by the severe stenosis degree of 85% across all three moderate stenosis degrees. This flow disturbance results in a jet impingement that varies in length with respect to the contralateral carotid stenosis degree. This stenosis results in differing carotid embolus recruitment from the cardiogenic source and altered downstream embolus dynamics.

Animation titled: ***S6_Movie.mp4*** compares two stenosis models where there is a mild 10% stenosis degree in the left carotid and a moderate degree of 50% in the right carotid presented in the left column and a severe stenosis degree of 85% in the right carotid presented in the right column. This animation compares flow rate directioj and magnitude within the communicating arteries that make up the collateral cerebral cirulation. We can see distinct differences between these cases in: (1) higher flow rates within the posterior and anterior region for the severe case (specifically the left internal carotid extension member, the right p1 connector, and the AcoA), (2) flow direction within the same vessels re-route towards the severe-hemisphere from the 10L50R case to the 10L85R with a decrease in the right internal carotid extension flow rate. These characteristics of collateral flow routing and magnitude changes allow for increased contralateral movement and non-intuitive movement within certain cases. This animation acts supplements Figure 9 showing the flow pathways that allow for embolus routing from the hemisphere ipsilateral to the mild/moderate stenosis to the contralateral, severe carotid stenosis hemisphere.

S3 Mathematical model details for hemodynamics simulation

Here we present the foundational equations for the stabilized finite element hemodynamic simulations. Blood was modeled as a Newtonian fluid with a bulk density of 1.06 g/cc and viscosity of 4.0 cP. whose momentum and mass balance obeys the standard Navier-Stokes and continuity equations stated in Eq. S1 and S2 as stated below:

$$\rho_f \left(\frac{\partial \mathbf{u}}{\partial t} + (\mathbf{u} \cdot \nabla \mathbf{u}) \right) = -\nabla p + \nabla \cdot \tau + \rho_f \mathbf{g} \tag{S1}$$

$$\nabla \cdot \mathbf{u} = 0 \tag{S2}$$

The term τ represents the viscous stresses in blood, which is modeled as per a linear Newtonian stress vs strain rate relation as follows:

$$\tau = 2\mu_f \mathbf{D}(\mathbf{u}) = \mu_f(\nabla\mathbf{u} + \nabla\mathbf{u}^T) \tag{S3}$$

These equations are solved using matrix systems of equations obtained by decomposing a stabilized Petrov Galerkin finite element variational formulation of these equations over a mesh composed of linear tetrahedral elements. This variational formulation is stated in Eq. S4 as follows:

$$\begin{aligned}
&\rho_f\left(\mathbf{w},\frac{\partial\mathbf{u}}{\partial t}\right)_\Omega + \rho_f(\mathbf{w},(\mathbf{u}\cdot\nabla)\mathbf{u})_\Omega + 2\mu_f(\mathbf{D}(\mathbf{w}),\mathbf{D}(\mathbf{u}))_\Omega \\
&-\rho_f(\mathbf{w},\mathbf{b})_\Omega - (\nabla\cdot\mathbf{w},p)_\Omega + (q,\nabla\cdot\mathbf{u})_\Omega + (q,\mathbf{u}\cdot\mathbf{n})_\Gamma \\
&-\underbrace{\left(\mathbf{w},\mathbf{h}_{\Gamma_{\Gamma_e}\in\Gamma_B}\right)}_{\text{traction boundaries}} + \underbrace{(\mathbf{w},\mathbf{h}_{0D})_{\Gamma_B}}_{\text{outflow boundaries}} + \sum_{e=1}^{N_{el}}\left[\underbrace{\left(\tau_{\text{supg}}\ \left(\mathbf{u}^h\cdot\nabla\right)\mathbf{w}^h,\mathcal{R}^h\right)_{\Omega,e}}_{\text{SUPG stabilization}} + \underbrace{\left(\tau_{pspg}\nabla q^h,\mathcal{R}^h\right)_{\Omega,e}}_{\text{PSPG stabilization}}\right] = 0
\end{aligned} \tag{S4}$$

In these equations, $\mathbf{u}$, p are blood flow velocity and pressure, $\mathbf{w}$, q are the two test functions employed in the finite element solver, μ_f is the averaged blood viscosity, ρ_f is the averaged density of blood, $\mathbf{g}$ designates gravity, Ω denotes the computational domain of the arterial network, Γ and the subscripts of Γ denote the boundary faces of the computational domain, $\mathcal{R}^h$ is the residual of the momentum equation, and $\tau_{supg/pspg}$ are the SUPG stabilization and PSPG stabilization factors used for convection and pressure stability respectively. $\mathbf{h}_{0D}$ represents the terms used to integrate the boundary conditions at the outlets and inlets of the arterial network; while $\mathbf{h}_{\Gamma_{\Gamma_e}\in\Gamma_B}$ represents the other boundary conditions that may be necessary to run physiologically realistic flow simulations.

S4 Mathematical model details for embolus dynamics simulation

Embolic particle simulations were conducted using a custom-modified version of the Maxey-Riley equation stated in Eq.S5 as below:

$$\begin{aligned}
m_p\frac{d\mathbf{v}_p}{dt} &= \underbrace{\frac{1}{2}\rho_f C_D\left(\pi R_p^2\right)\|\mathbf{u}-\mathbf{v}_p\|(\mathbf{u}-\mathbf{v}_p)}_{\text{drag force}} + \underbrace{C_{sl}\rho_f R_p^2\frac{\sqrt{\mu_f}}{\sqrt{\rho_f\|\omega\|}}\left[(\mathbf{u}-\mathbf{v}_p)\times\omega\right]}_{\text{lift force (shear-gradient)}} \\
&+ \underbrace{\mathcal{V}_p(-\nabla p+\nabla\cdot\tau)}_{\text{fluid stresses, undisturbed flow}} + \underbrace{C_{\text{am}}\frac{\rho_f\mathcal{V}_p}{2}\left[\frac{D\mathbf{u}}{Dt}-\frac{d\mathbf{v}_p}{dt}\right]}_{\text{added mass}} + \underbrace{(m_p-\rho_f\mathcal{V}_p)\,\mathbf{g}}_{\text{buoyancy}} + \underbrace{\mathcal{I}(\mathcal{P},\mathcal{W})\left[\Delta\mathbf{v}_p\left(e_{\text{lub}}\right)\right]}_{\text{elastohydrodynamic wall-collisions}}
\end{aligned} \tag{S5}$$

where m$_p$ notes the particle mass, $\mathcal{V}_p$ is the particle volume, R$_p$ is the particle radius, and $\mathbf{v}_p$ is the translational particle velocity. C$_a m$ is the added mass coefficient, C$_D$ is the drag coefficient, and C$_s l$ denotes the shear-gradient lift force. $\omega = \nabla$ x $\mathbf{u}$ is the vorticity of the flow where the particle is located. $\mathcal{I}(\mathcal{P},\mathcal{W})$ is an indicator that has a value of 1 when a particle $\mathcal{P}$ comes into contact with the artery wall, the value of this function is 0 otherwise. Next, the contribution of the particle-wall collision to the change in particle velocity is denoted by the term $[\Delta\mathbf{v}_p(\mathrm{e}_{lub})]$, which accounts for elastohydrodynamic lubrication effects at the collision point. The term e_{lub} is the restitution coefficient. Figure S1 illustrates the seeding locations of embolus across the carotid and cardioembolic releases and the offset distance between the arterial wall and embolus that is equivalent to the radius of the embolus.

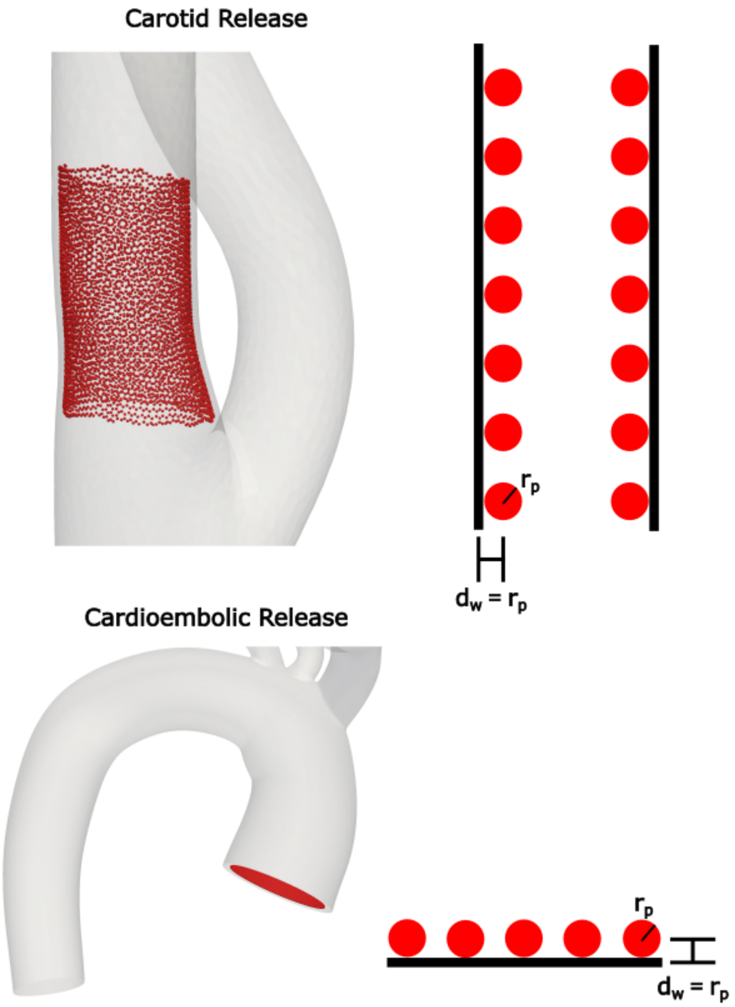

Fig S1: *Illustrates how particles are seeded at both the cardioembolic and carotid sources. Embolus are offset from the wall by factor d_w that is equal to the radius of embolus considered. For all cases considered in this study, a particle size of 500 microns was used with a d_w of 250 microns across all releases.*

S5 Length of stenosis region

The length of the stenosed region across the span of the carotid artery was calculated using the equation below:

$$Length = \alpha * (1 + NASCET) * D_{CCA} \tag{S6}$$

where α is a constant factor of 0.65 used across all stenosis applications as described in the literature. NASCET is the associated NASCET stenosis degree applied for that model. D_{CCA} is the diameter of the undisturbed common carotid artery at that ipsilateral carotid artery. This relation, for a chosen NASCET severity, was used to reduce the segmentation diameters and re-loft the models to create stenosed carotid artery models.

S6 Complete distribution dataset for emboli across all vessels

For each simulation, the total proportion of embolus samples released was calculated across each outlet of our heart-to-brain model. All embolus source-to-distribution statistics reported in the manuscript were derived from these distributions. While in the manuscript the focus was on contralateral trans-hemispheric distribution, the complete set of embolus distribution across all vessel outlets individually is not included in the main manuscript. We provide a visual depiction of this complete dataset for each embolus release source as additional reference in the supplementary figures below. The vessel outlets included the anterior cerebral arteries (ACAs), the posterior cerebral arteries (PCAs), the middle cerebral arteries (MCAs), the subclavians, the external carotids, and the descending aorta (DA).

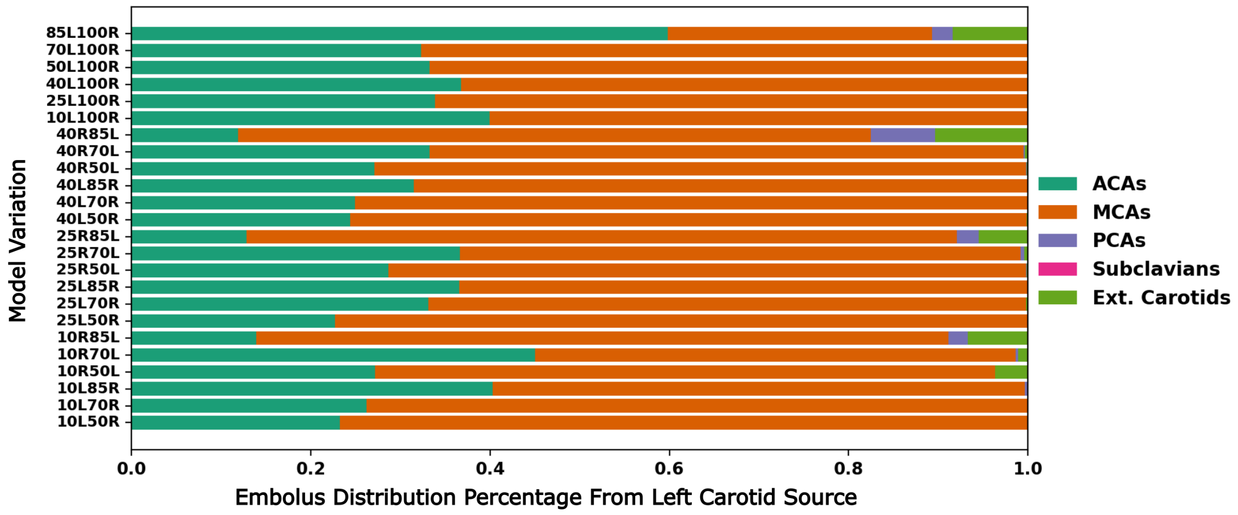

Fig S2: *Complete simulated embolus distribution data across all left carotid embolus releases, for all possible vessel outlets in the heart-to-brain pathway modeled for this study.*

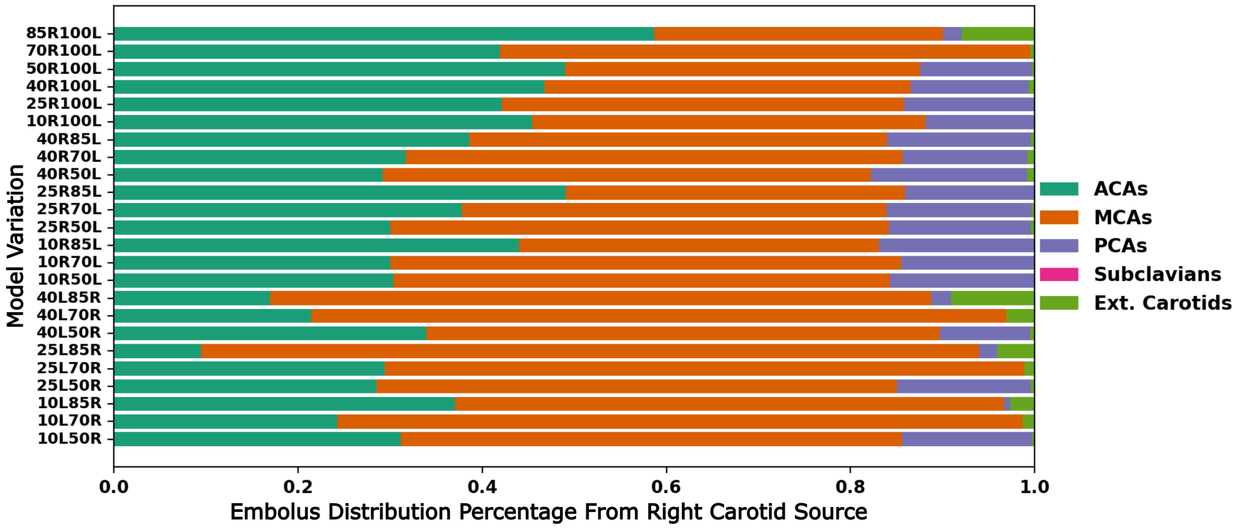

Fig S3: *Complete simulated embolus distribution data across all right carotid embolus releases, for all possible vessel outlets in the heart-to-brain pathway modeled for this study.*

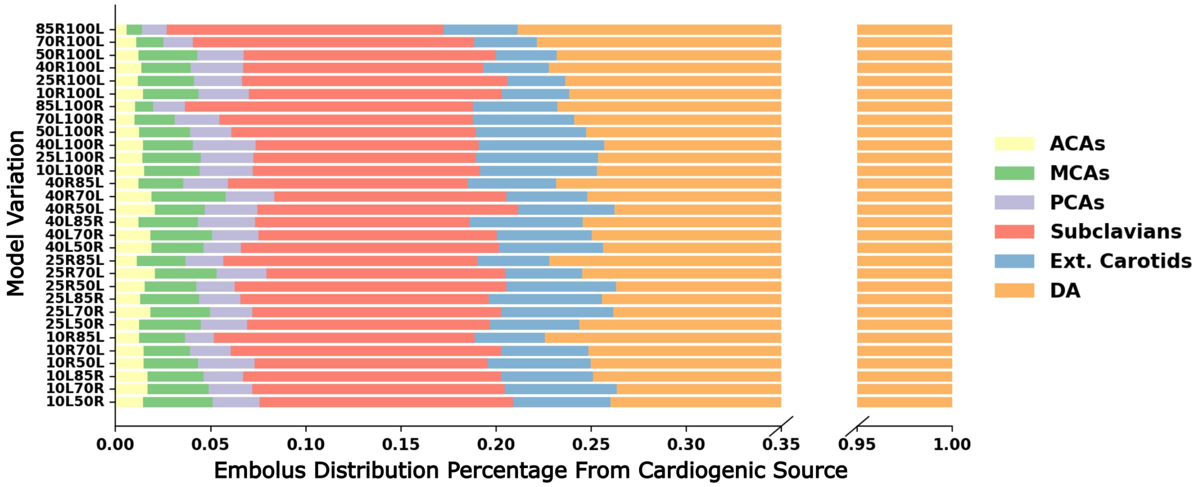

Fig S4: *Complete simulated embolus distribution data across all cardiogenic embolus releases, for all possible vessel outlets in the heart-to-brain pathway modeled for this study.*
